# Genotype-specific incidence and clearance rates of human papilloma virus (HPV) infection in HIV-infected women from Pune, India

**DOI:** 10.1186/1471-2334-12-S1-O2

**Published:** 2012-05-04

**Authors:** Arati Mane, Amit Nirmalkar, Arun R Risbud, Sten H Vermund, Sanjay M Mehendale, Vikrant V Sahasrabuddhe

**Affiliations:** 1 National AIDS Research Institute, Pune, India; 2 Vanderbilt University Institute for Global Health, Nashville, USA; 3 National Institute of Epidemiology, Chennai, India

## Background

Information on HPV genotype-specific incidence and clearance is important to better inform the natural history of cervical neoplasia in context of HIV/AIDS. We conducted a cohort study among HIV-infected women in Pune, India to estimate the genotype-specific HPV incidence and clearance rates.

## Methods

HIV-infected women underwent baseline and annual follow-up visits for cervical cancer screening and collection of cervicovaginal samples for detection of 37 HPV genotypes by Linear Array polymerase chain reaction (PCR) assay.

## Results

A total of 215 eligible participants were followed for a median time of 11 months (range: 8-23 months) with a follow-up period of 223 person-years. Of the 104/215 (48.4 %) HIV-infected women who were HPV-negative at baseline, 12 women were newly detected with HPV at follow-up visit reflecting an incidence rate of 5.4 per 100 person-years. Type-specific incidence rates ranged between 0.45-3.42 per 100 person-years for carcinogenic HPV types and between 0.45-1.79 per 100 person-years for other HPV types. Of the 111/215 (51.6 %) women with HPV at baseline, 21 women cleared all types, reflecting a clearance rate of 9.4 per 100 person-years. Type-specific clearance rates ranged between 0.45-4.48 per 100 person-years for carcinogenic HPV types and between 0.45-4.04 for other HPV types.

## Conclusions

This study adds to the scant global data of natural history of HPV infection in HIV-infected women. Knowledge of incidence and clearance rates can inform cost effectiveness and decision analysis models for estimating effectiveness of HPV vaccination and screening strategies for cervical cancer prevention for HIV-infected women.

